# Incidence and Variants of Posterior Arch Defects of the Atlas Vertebra

**DOI:** 10.1155/2013/957280

**Published:** 2013-09-10

**Authors:** Sebastian Guenkel, Sladjana Schlaepfer, Sonja Gordic, Guido A. Wanner, Hans-Peter Simmen, Clément M. L. Werner

**Affiliations:** ^1^University Hospital Zurich, Division of Trauma Surgery, 8091 Zurich, Switzerland; ^2^University Hospital Zurich, Department of Radiology, 8091 Zurich, Switzerland

## Abstract

In order to describe the incidence and existing variants of congenital anomalies of the atlas vertebrae in a Caucasian population, we examined 1069 CT scans of the upper cervical spine. We found 41 cases with altered atlas vertebrae, representing 3.8% of all analyzed patients. With 83% of all found anomalies, the predominant type is characterized by a small dorsal cleft (3.2% of all patients). Rare varieties feature unilateral or bilateral dorsal arch defects, combined anterior and posterior clefts (0.2% of all patients) or total erratic atlas vertebra malformation (0.1% of all patients). Atlas arch defects are found nearly 4% at the time. Most anomalies affect the posterior arch, whereas the anterior arch or both are rarely affected. Totally irregular C1 vertebrae are extremely infrequent.

## 1. Introduction

Atlas arch anomalies are found mostly coincidentally. The predominant defect involves the posterior arch [1–4]. Currarino et al. proposed 5 types of atlas posterior arch defects referring to Torklus [2, 5]. The anomalies vary from unifocal clefts to total absence of the posterior arch and posterior tubercle. Less common are anterior atlas arch defects and the combination of both [3, 4].

Accompanying anomalies include an enlarged anterior arch, cephalad elongation of the spinous process of the axis, and a dense fibrous membrane forming a posterior atlanto-occipital membrane [2]. These altered anatomical findings exhibit natural adaption in order to maintain stability and function. In cervical spine trauma, profound knowledge of congenital atlas defects is crucial. Malformations, where C1/C2 junction might be compromised, have to be distinguished from fractures. 

We therefore conducted this study to further describe defects of the atlas vertebra and to estimate their incidence. The found anomalies were examined and grouped.

## 2. Materials and Methods

The institutional review board approved this retrospective study waiving the need for patient consent.

We retrospectively reviewed 1069 consecutive cervical CT scans from our trauma database. Indication for the CT scans was adequate trauma with the risk of a cervical spine injury and/or the presence of clinical symptoms. Cases with atlas fractures, severe degeneration, and previous operations were excluded. Multiplanar CT reconstructions (axial and sagittal) in 1.5 mm slices were evaluated (Siemens Somatom Definition Dual Source). For each subject, anatomical alteration of the atlas vertebra of any kind was analyzed. The CT scans were examined by 2 independent reviewers. The atlas anomalies were studied, and described. Data were collected and descriptive statistical analysis was performed using SPSS software (version 20).

## 3. Results

1069 patients were eligible for the study. We reviewed 255 cervical spine CT scans, 3 cervical and thoracic spine CT scans, 28 cervical, thoracic and lumbar spine CT scans, 9 neck and thorax CT scans, and 774 whole body (neck, thorax, abdomen, and pelvis) CT scans. 13 patients were excluded because of severe cervical spine degeneration, 7 because of atlas fractures and 2 because of previous operations on the atlas. One patient was excluded with an untypical small sclerotic dorsal discontinuity, nondistinctive for a congenital nonfusion or old fracture. 

**Figure 1:**
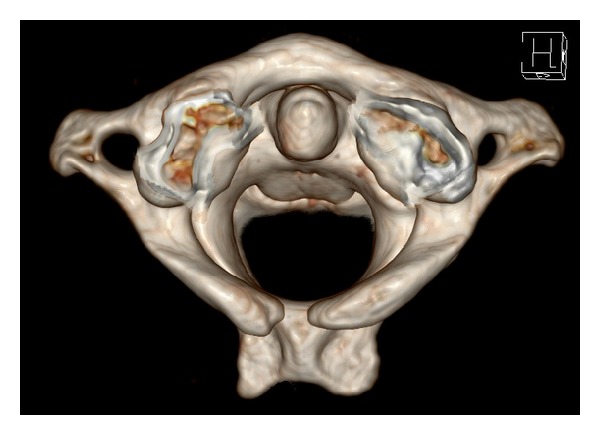
Typical dorsal arch defect (according to Currarino et al. type A [2]).

In the 1069 analyzed patients, we found 41 cases of atlas arch defects. This represents 3.8% of all patients. Of the 41 found anomalies, 38 cases presented a dorsal arch defect (92.7% of all anomalies and 3.6% of all examined patients). Type A was predominant with 34 cases (82.9% of the malformations and 3.2% off all patients, resp.).  Figure 1 shows a typical example of type A according to the classification of Currarino et al. [2].

Types B and C were both found in 2 patients (each 4.8% of all anomalies and 0.2% of all patients, resp.). No type D or E was found. 

**Figure 2:**
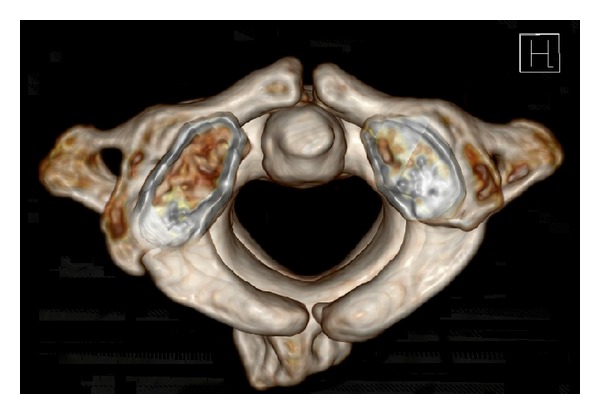
Bipartite spondyloschisis.

A bipartite spondyloschisis was present in 2 cases of our cohort (4.8% of all atlas arch defects, 0.2% of all patients, resp., e.g., see Figure 2).

One patient showed a total irregular form of the atlas vertebra. This erratic form represents only 2.4% of all atlas arch defects and 0.1% of all examined patients. 

Four patients suffered an accompanying fracture of another cervical spine vertebra (one type A with Anderson type I dens fracture and a dislocated fracture of C5 spinous process, another type A with incomplete C7 burst fracture, one type A with C7 spinous process fracture, and one type C with Anderson type III dens fracture). 

## 4. Discussion

### 4.1. Development of Congenital Atlas Arch Defects

The embryological development is essential for understanding congenital atlas arch defects. The body of atlas vertebra derives from the primitive fourth occipital and first cervical sclerotomes. Three or more ossification centers form the atlas [1]. Usually one midline center builds the anterior arch in the seventh week of gestation. Sometimes the anterior arch derives from two different origins. At the same time, two ossification centers form the lateral masses [6]. There might be an additional ossification center representing the posterior tubercle. Unification between the ossified atlas parts occurs at five to nine years of age [7]. The ossification usually proceeds perichondrally. 

The pathogenesis of atlas abnormalities is not yet fully known. Proposed explanations are a local disorder in dorsal occlusion of the neural tube during early embryologic evolution [1, 8, 9]. Subsequent dysfunction of chondrification or ossification is discussed [7, 8, 10].

### 4.2. Incidence

In this study, we could show an incidence of 3.8% of atlas arch defects. The incidence in the literature varies between 0.69 and 4% [1–5, 11, 12]. It seems that in Caucasian population congenital atlas arch defects are more frequent than in Asian population [1, 3, 4]. Consistent with the literature, in our patient cohort posterior arch defects are predominant. The most frequently found atlas anomaly is accompanied by a relatively small dorsal cleft, according to Currarino et al. type A [2–4]. Similar to the findings of the published literature, anterior arch defects and bipartite spondyloschisis with a combination of anterior and posterior atlas arch defects are rare. We found only 2 cases out of the 1069 examined patients with anterior and posterior defects. Irregularly shaped atlas deformities seem to be exceedingly infrequent with less than 1 : 1000. 

## 5. Conclusion

A variety of congenital atlas arch defects exist. The knowledge about preexisting malformations and their clinical and radiological appearance is important in order to direct diagnostic workup and to identify patients at risk. In the examined patient cohort, almost 4% presented with congenital atlas arch defects. Consistent with the literature the predominant type found in this study is associated with a small posterior arch defect (3.2% of all patients). Rarities are bipartite spondyloschisis and atlas bodies with total irregular defects.
